# Epidemiological and clinical burden of *EGFR* Exon 20 insertion in advanced non-small cell lung cancer: A systematic literature review

**DOI:** 10.1371/journal.pone.0247620

**Published:** 2021-03-08

**Authors:** Heather Burnett, Helena Emich, Chris Carroll, Naomi Stapleton, Parthiv Mahadevia, Tracy Li

**Affiliations:** 1 Evidera, Montreal, QC, Canada; 2 Evidera, London, United Kingdom; 3 The University of Sheffield, Sheffield, United Kingdom; 4 Janssen Global Services, Raritan, NJ, United States of America; Osmania University, Hyderabad, INDIA

## Abstract

**Objectives:**

The burden of epidermal growth factor receptor (*EGFR*) exon 20 insertion mutation (Exon 20ins) in non-small cell lung cancer is not well understood. A systematic review was conducted to identify evidence on mutation frequency, prognostic impact, clinical, patient-reported, and economic outcomes associated with Exon 20ins.

**Materials and methods:**

Searches were conducted in Embase and Medline and supplemented with recent conference proceedings. Included studies were not limited by intervention, geography, or publication year.

**Results:**

Seventy-eight unique studies were included; 53 reporting mutation frequency, 13 prognostic impact, 36 clinical outcomes, and one humanistic burden. No economic burden data were identified. The frequency of Exon 20ins mutation ranged from 0.1% to 4% of all NSCLC cases and 1% to 12% of all *EGFR* mutations. Data on the prognostic impact of Exon 20ins were heterogeneous but highlighted poorer outcomes in patients with Exon 20ins mutation compared with patients with other *EGFR* mutations and *EGFR* wildtype across a wide range of therapies and treatment lines. Comparative evidence on the clinical efficacy and safety of currently available therapies were limited, as were sample sizes of studies reporting on real-world effectiveness. Nine single-arm trials and 27 observational studies reported clinical outcomes for patients with Exon 20ins. Trends towards better survival and response were observed for chemotherapy compared with TKIs as first-line treatments. For subsequent treatment lines, novel targeted therapies provided encouraging preliminary responses while results for chemotherapy were less favorable. Limited safety data were reported. One conference abstract described the symptom burden for Exon 20ins patients with fatigue and pain being most common.

**Conclusion:**

Findings of the systematic review show a high unmet need for safe and efficacious treatments for patients with Exon 20ins as well and need for further evidence generation to better understand the patient-level and economic impact for these patients.

## Introduction

Approximately 30% of non-small cell lung cancer (NSCLC) tumors harbor a mutation in the epidermal growth factor receptor (*EGFR*) gene, with geographical variation in rates reported to be highest in Asia (38%) and lowest in Europe (14%) [1]. Approximately 85%-90% of *EGFR* mutations comprise Exon 19 deletion and L858R point mutations of Exon 21 (classical *EGFR* mutations) [2, 3], while the remaining 10%-15% comprise uncommon mutations, including Exon 20 insertion (Exon 20ins) mutation (4–12%), L861Q (3%), G719X (2%) and S768I (1%) [4, 5]. The Exon 20ins mutation is the third most common type of *EGFR* mutation in NSCLC, after Exon 19 deletions and Exon 21 L858R point mutations [2, 4, 5]. The expanded use of next-generation sequencing (NGS) in clinical practice, and thus an improved ability to identify rare variants, has led to an increase in the incidence of uncommon *EGFR* mutations, including Exon 20ins [6]. The growing incidence and high heterogeneity (with potentially variable sensitivity to *EGFR* blockage) of Exon 20ins mean this uncommon mutation is increasingly clinically relevant [6, 7].

The population of patients with NSCLC and Exon 20ins is not well recognized, and no specific treatment recommendations have been made in European (European Society for Medical Oncology [8]) or US (National Comprehensive Cancer Network [9]) clinical guidelines [8, 9]. Patients are usually treated with chemotherapy or *EGFR* tyrosine kinase inhibitors (TKIs) [10], although, *EGFR* Exon 20 mutations (including Exon 20ins) have been associated with resistance/insensitivity to currently available TKIs [3–5, 11]. The US FDA recently granted Breakthrough Therapy Designations for two investigational targeted therapies (amivantamab [JNJ-372] [12] and mobocertinib [TAK-788] [13]) for the treatment of patients with metastatic NSCLC with *EGFR* Exon 20ins mutation whose disease has progressed on or after platinum-based chemotherapy. Even as the treatment landscape for NSCLC Exon 20ins rapidly evolves, clinical outcomes and the optimal treatment choice remain poorly understood for this patient population.

Global variations in the frequency of mutation and prognostic impact of Exon 20ins, as well as the humanistic and economic burden for this specific population, are also not well understood or reported. Thus, a systematic literature review (SLR) was conducted to provide a comprehensive summary of the available evidence on the burden of Exon 20ins in NSCLC. Evidence was collated on: 1) the global frequency of Exon 20ins, 2) the prognostic impact of Exon 20ins compared to other *EGFR* mutations and the association between other patient factors and Exon 20ins, 3) treatment patterns and clinical outcomes in patients with Exon 20ins, and 4) the cost, resource use, and humanistic burden of Exon 20ins. Findings from the SLR will help to consolidate the current body of evidence and identify gaps for future research.

## Methods

The SLR was conducted using rigorous methodology and in accordance with the Cochrane Handbook for Systematic Reviews of Interventions [14] and the Preferred Reporting Items for Systematic Reviews and Meta-Analyses (PRISMA) guidelines [15] with regards to the methods used to search, identify, review, and summarize the available evidence.

### Identification and selection of studies

The database searches were conducted in MEDLINE and Embase via Ovid on September 10, 2019 and were not limited by intervention, study design, geography, or publication year. The search strategy is presented in S1 Table. In addition to the databases 2019 and 2020 proceedings from ESMO, World Conference on Lung Cancer (WCLC), and American Society of Clinical Oncology (ASCO) conferences were searched. Bibliographies of published systematic reviews and/or meta-analyses of relevant studies that were identified during the database searches were also reviewed to identify additional, relevant publications.

Pre-defined inclusion and exclusion criteria were used to evaluate the titles and abstracts of the records identified by the searches in the first level of review. Full-text articles of abstracts that were deemed relevant during the first level of review were retrieved and reviewed. Title and abstract screening were conducted by a single researcher, while full-text screening was conducted by two independent researchers with disagreements resolved by a third researcher. The pre-defined selection criteria based on the populations, interventions and comparators, outcomes and study design (PICOS) framework are shown in Table 1.

**Table 1:** PICOS selection criteria.

	Selection Criteria
**Population**	Adults (≥18 years) with advanced/metastatic NSCLC and *EGFR* Exon 20ins mutation
**Interventions/Comparators**	Any/none
**Outcomes**	Epidemiology: Incidence, prevalence, and frequency of mutation (by subtype, race, age, or other patient subgroups)
	Prognostic value of Exon 20ins mutation in terms of OS, PFS, and ORR
	Clinical burden: Efficacy (OS, PFS, ORR) and safety
	Humanistic burden: HRQoL, utilities, and symptoms
	Economic burden: Cost (direct and indirect), resource use, and cost-effectiveness outcomes
**Study Design**	Epidemiological (population/registry-based)
	Observational cohort studies (prospective or retrospective)
	Cross-sectional studies
	RCTs
	Prospective, interventional studies (non-randomized trials)
**Geography**	No limit
**Language**	No limit
**Sample size**	Excluded epidemiology studies wherein the number of NSCLC patients genotyped for Exon 20 insertion were <250
	Excluded prognostic impact and clinical burden studies wherein the number of Exon 20 insertion patients were <10

Abbreviations: *EGFR* = epidermal growth factor receptor; ins = insertion; NSCLC = non-small cell lung cancer; ORR = objective response rate; OS = overall survival; PFS = progression-free survival; RCT = randomized controlled trial; TKI = tyrosine kinase inhibitor

### Data extraction

Standardized data extraction tables developed in Microsoft Excel® were used to capture and present key evidence from each of the studies that met the pre-defined inclusion and exclusion criteria. Data for all topics were extracted by one independent researcher and validated by a second researcher to ensure their accuracy.

## Results

### Study selection

**Fig 1:**
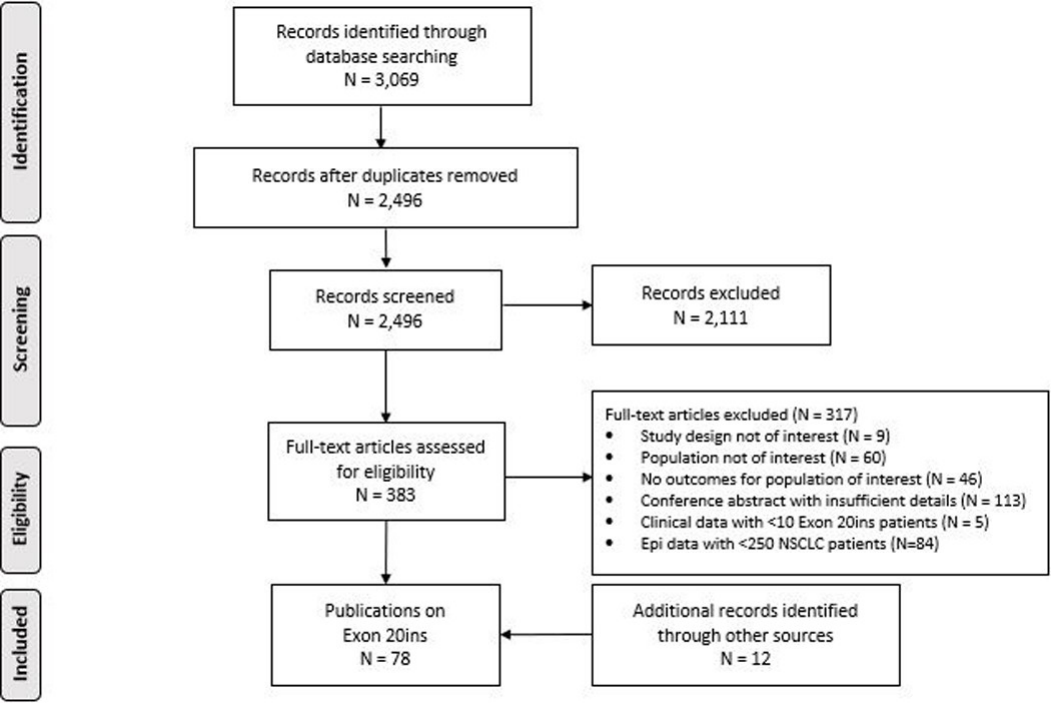
PRISMA diagram.

The electronic searches yielded 2,499 unique records for title and abstract screening (Fig 1).

A total of 386 records were screened at the full-text level, with most excluded citations being conference abstracts that reported limited information (113/317). After applying the PICOS criteria and conducting searches of conference abstracts, 78 articles were included: 53 reporting the frequency of Exon 20ins, 13 reporting on the prognostic impact, 36 reporting clinical outcomes, and one reporting on the humanistic burden (S2 Table). No eligible articles reported on the economic impact of Exon 20ins.

### Frequency of mutation

#### Study characteristics

Studies reporting the frequency of the Exon 20ins mutation varied with regards to population ethnicity or global region assessed, with most studies reporting data from Asia Pacific (n = 22 studies), followed by Europe (n = 12), the US (n = 10), and Latin America (n = 7). Most studies were single-center (n = 38), while 14 were multi-center and one was a meta-analysis.

The most commonly reported mutation test methodology was real-time polymerase chain reaction (RT-PCR)/Sanger (n = 16), followed by RT-PCR/Cobas (n = 6), Amplification Refractory Mutation System™ (ARMS; n = 6), NGS/capture-based comprehensive genomic profiling (n = 4), and mass spectroscopy genotyping (n = 2). Twenty studies did not report the methodology used for mutation testing.

#### Frequency of mutation

Across countries, the frequency of *EGFR* Exon 20ins ranged from 0.1%–4.0% of all NSCLC cases and from 1%–12% of all *EGFR* mutations (Table 2).

**Table 2:** Global Exon 20ins rates.

Region	Frequency of *EGFR* Exon 20ins (%)			
	# Studies	NSCLC	# Studies	*EGFR*m Positive
US	9	0.5–2.6%	7	5–12%
Latin America	7	1.3–2.1%	5	5–8%
Europe	13	0.3–1.3%	10	4–12%
Asia Pacific	28	0.1–4.0%	16	1–5%
South Asia	5	0.3–3.4%	4	1–4%
South East Asia	4	0.1–2.4%	2	2–3%
Japan	4	1.8–2.4%	2	2–5%
China	9	0.3–2.9%	7	2–5%
Taiwan	3	1.3–4.0%	2	3–4%

Abbreviations: *EGFR*m = epidermal growth factor receptor mutation; Exon 20ins = exon 20 insertion mutation; NSCLC = non-small cell lung cancer

The highest frequencies were reported for the Asia Pacific region (0.1%–4.0% among all NSCLC cases and 1%–5% of *EGFR*m tumors) and the US (0.5%–2.6% among all NSCLC cases and 5%–12% of *EGFR*m tumors).

Ten studies reported patient-level factors that significantly impact the frequency of Exon 20ins [4, 5, 11, 16–22]. The mutation was found to be more common in women (47% of Exon 20ins vs. 28% of *EGFR* wildtype (WT), p = 0.003) [19], Asian patients (15% of Exon 20ins vs. 4% of *EGFR* WT, p = 0.02) [5], never-smokers (56% of Exon 20ins vs. 20% of *EGFR* WT, p<0.0001 [5] and 67% of Exon 20ins vs. 26% of other uncommon *EGFR* mutations, p<0.01) [20], older patients (p = 0.01, p = 0.032 vs. Exon 19 deletion/L858R [11, 19]), and those with adenocarcinoma histology (100% for Exon 20ins vs. 76% of G719X, 82% of L861Q/P, 89% of L858R, and 93% of Exon 19 deletion [p-values not reported]) [22].

### Prognostic impact

#### Study characteristics

Studies that reported on the prognostic impact of Exon 20ins varied significantly with regards to the genotypes compared, line and type of therapy received, and patient ethnicity. The majority of studies were retrospective chart reviews (n = 10), with three exceptions: one post-hoc analysis of trial data (LUX-Lung 2, LUX-Lung 3, and LUX-Lung 6) [21] and two prospective registries [23, 24]. Most studies were conducted in Asia (n = 8), with the remainder in the US (n = 3), Europe (n = 1), and Latin America (n = 1). Half of the studies evaluated TKI-treated populations and half evaluated mixed-treatment populations (i.e. chemotherapy and/or TKIs). A majority evaluated populations receiving mixed lines of therapy, although some specifically evaluated first-line (n = 3) and subsequent lines (n = 2) of therapy.

The genotypes most frequently assessed alongside Exon 20ins with regards to outcomes of interest were classic mutations (Exon 19 deletion and L858R) and the T790M mutation, evaluated in nine and six studies, respectively. Other mutations assessed alongside Exon 20ins, such as Exon 18 mutations, other uncommon mutations, and *EGFR* wildtype (wt) were not reported frequently enough to draw conclusions across the literature.

#### Exon20ins vs. classic mutations

Nine studies demonstrated a survival and/or response benefit for patients with classic *EGFR* mutations (Exon 19 deletion and L858R) compared with Exon 20ins [11, 19, 20, 25–30]. OS in patients with the classic mutations was at least double that of patients with Exon 20ins in nearly all studies comparing these groups [11, 20, 26, 30, 31]. Median OS ranged from 4.8–16.8 months in the Exon 20ins groups (range 11–84 patients) and 17.3–31.6 months in the groups with classic mutations (range 186–1,816 patients) (Table 3).

**Table 3:** Summary of prognostic impact of Exon20 insertions compared to other genotypes.

	Range Median OS	Range Median PFS	Range ORR
	(months)	(months)	(%)
**TKIs**			
Exon 20ins	4.8–19	1.4–3.0	0–20%
	6 studies	8 studies	7 studies
	177 patients (range 11–67)	183 patients (range 11–67)	194 patients (range 11–67)
Classic *EGFR*m (del 19 or L858R)	19.6–27.7	8.5–15.2	27.4–84%
	3 studies	3 studies	5 studies
	501 patients (range 37–278)	501 patients (range 37–278)	1193 patients (range 37–692)
T790M	13.5–27.7	1.0–2.9	0–25%
	3 studies	3 studies	4 studies
	67 patients (range 14–30)	67 patients (range 14–30)	114 patients (range 14–47)
Wild-type	10.4–21	2	16.50%
	2 studies	1 study	1 study
	990 patients (range 20–88)	1261 patients (range 15–39)	1261 patients (range 20–102)
**TKI and/or Chemotherapy**			
Exon 20ins	14.6–26	4.8–6.0	5.1–28%
	4 studies	2 studies	2 studies
	233 patients (range 258–1,816)	99 patients (range 15–39)	172 patients (range 20–102)
Classic *EGFR*m (del 19 or L858R)	17.3–31.6	No study	0 studies
	3 studies		
	2106 patients		
T790M	12.3	8.2	0 studies
	1 study	1 study	
	9 patients	9 patients	
Wild-type	0 studies	0 studies	0 studies

Abbreviations: del = deletion; *EGFR* = epidermal growth factor receptor; ins = insertion; LOT = line of therapy; m = mutation; ORR = objective response rate; OS = overall survival; PFS = progression-free survival; TKI = tyrosine kinase inhibitor; WT = wild type

*Data is for a subtype of Exon 20ins patients with a specific insertion (V769_D770insASV (2307–2308) GCCAGCGTG)

One exception was a retrospective study from Memorial Sloan Kettering Cancer Center in New York, which reported comparable median OS for Exon 20ins (46 patients) vs. classic mutations (258 patients); 26 versus 31 months (p = 0.53) [11]. The majority of patients in this study received chemotherapy followed by erlotinib, whereas most other studies evaluated TKIs. In a retrospective case series from National Taiwan University, median OS in 43 patients with Exon 20ins receiving first-line pemetrexed approached that of 1,064 patients with classic *EGFR* mutations receiving first-line chemotherapy or TKIs; 28 months versus 31.6 months, whereas 16 Exon 20ins patients treated with first-line TKI-containing regimens had much lower survival (16.8 months) [32]. Most studies evaluated mixed lines of therapy, so the impact across individual lines of therapy is unclear.

Notably, the presence of an Exon 20ins mutation alone appears to have poorer outcomes than Exon 20ins with concomitant classic *EGFR* mutations (del 19 and L858R). A retrospective case series conducted in in Latin America showed that Exon 20ins alone (88 patients) was associated with significantly shorter survival compared to Exon 20ins with a concomitant classic *EGFR* mutation (32 patients); 14.6 vs.17.3 months (p = 0.017) [33].

The impact of Exon 20ins compared to classic *EGFR* mutations is even more pronounced for PFS, with median PFS 4.2 to 6.0 times longer in patients with classic mutations (range: 1.4–3.0 months [range 11–67 patients] vs. 8.5–15.2 months [range 37–278 patients]) [20, 26, 27]. Similarly, the included studies found a higher response rate for classic *EGFR* mutations compared to Exon 20ins. Studies examining PFS and objective response rate (ORR) evaluated TKIs across mixed lines of therapy, and therefore the relationship in the context of treatment and line of therapy cannot be examined.

#### Exon 20ins vs. T790M

Four studies compared OS for patients with Exon 20ins vs. T790M [21, 23, 27, 34], with most demonstrating a numerical but not statistically significant improvement in OS for patients with the T790M mutation. Studies wherein patients received TKIs across mixed lines of therapies showed a survival benefit for patients with T790M (range 14–30 patients) compared to Exon 20ins (range 5–67 patients); 14.9–27.7 months vs. 9.2–12.5 months [21, 27]. In contrast, one study of TKIs given in subsequent lines of therapy showed no difference in OS between Exon 20ins (29 patients) vs. T790M (23 patients); 12.9 vs. 13.5 months [23]. This study included patients evaluated from 2005 to 2014, prior to approval of osimertinib. Therefore, it is possible that line of therapy may cause these differences.

One retrospective cohort study at a single center in India in patients receiving a combination of TKI and chemotherapy demonstrated longer median OS in patients with Exon 20ins (15 patients) compared to those with T790M (9 patients); 15.8 months vs. 12.3 months [34].

Findings on PFS were similarly inconclusive for Exon 20ins compared to T790M, with three studies showing longer PFS for patients with T790M and one showing shorter PFS. Among the three studies reporting a longer PFS for T790M, the difference ranged from 0.2–2.2 months (range 15–29 Exon 20ins patients and 9–23 T790M patients) [21, 23, 34], while the fourth study in which patients received either erlotinib or gefitinib reported longer PFS for patients with Exon 20ins (67 patients) vs. T790M (30 patients); 3.0 months vs 1.0 months [31].

Studies presented varied conclusions on ORR. Two studies [23, 25] reported marginally higher response rates for the T790M patients compared with Exon 20ins patients; the first study [23] reported lower response rates for T790M patients (23 patients) compared with Exon 20ins patients (29 patients), and the second [25] observed no objective responses in either group. No study evaluated statistical significance between these mutation groups for this outcome, and therefore no conclusions could be drawn.

#### Exon 20ins vs. wildtype

Two studies [26, 35] compared outcomes for Exon 20ins (11 and 25 patients) versus *EGFR* WT (272 and 718 patients). In both studies, patients received erlotinib or gefitinib, with similar overall survival (OS) between groups (no statistically significant differences). One study compared progression-free survival (PFS) for Exon 20ins (11 patients) versus *EGFR* WT (272 patients), and found similar median PFS between groups;1.4 vs. 2.0 months, respectively [26]. Given the limited sample size of the Exon 20ins group compared to WT group, these findings should be interpreted while considering this imbalance.

### Clinical burden

#### Study characteristics

Similar to the studies reporting prognostic impact, studies that reported clinical outcomes for patients with Exon 20ins varied significantly with regards to study design, setting, line and type of therapy received, and sample size (Table 4).

**Table 4:** Overview of studies reporting the clinical burden of Exon20 insertions by line of therapy and drug class.

Author, Year (Trial Name)	Study Design	Population Description (Sample Size)	Study Setting (# centers)	Exon20 Insertion Sample Size	Treatments received	mOS, months (95% CI)	mPFS, months (95% CI)	ORR (95% CI)
**First-line treatment**								
Yang 2015 [21]	Post-hoc analysis of 3 trials^a^	Uncommon *EGFR*m-positive advanced (stage IIIb–IV) lung adenocarcinomas (75)	NR	23 (subgroup)	Afatinib	9.2 (4.1−14.2)	2.7 (1.8−4.2)	CR+PR: 8.7% (1.1–28.0)
Kate 2019 [34]	Retrospective observational	Uncommon *EGFR*m-positive advanced NSCLC (83)	India	15 (subgroup)	Oral TKI (erlotinib, gefitinib or osimertinib, 100%)	15.8 (6.2–25.3)	1.9 (0.3–3.5)	0%
Yasuda 2013 [36]	Retrospective observational	Patients with *EGFR* Exon 20ins mutated NSCLCs (19)	5 centers in Singapore (n = 1) and the US (n = 4)	19	Gefitinib or erlotinib	Range: 0.9–71.0	Range: 0.8–18.0	NR
Cardona 2018 [33]	Retrospective observational	Stage II-IV NSCLC with Exon 20ins mutation (88)	6 Latin American countries (Argentina, Colombia, Costa Rica, Ecuador, Panama, and Mexico)	88	TKI-based regimen (erlotinib, gefitinib, or afatinib, 31.7%) or platinum CT-based regimen (68.3%)	16.4 (14.4–18.3)	TKI (n = 28): 6.4 (5.0–10.1) CT (n = 60): 6.9 (5.4–8.3)	28%
Wang 2019 [37]	Retrospective observational	Patients with *EGFR* Exon 20ins insertion mutated NSCLC (165)	99 hospitals in 26 different regions in China	165	Platinum-based CT or *EGFR* TKIs (all generations)	NR	Platinum-based CT (n = 105): 6.4 (5.7–7.1) *EGFR* TKI (all generations, n = 23): 2.9 (1.5–4.3, p<0.001) *EGFR* (1gen, n = 14): 2.6 (0.8–4.4; p<0.001)	NR
DerSarkissian 2019 [10]	Retrospective observational (Flatiron Health database)	Treatment-naïve patients with advanced/ metastatic NSCLC with *EGFR* Exon 20ins (128)	US	128	CT, *EGFR* TKI, IO	All patients (n = 128): 16.2 (13.8–18.6) *EGFR* TKI only (n = 27): 7.1 (2.8–19.4) Any IO (n = 8): 6.1 (2.0–NR) CT (n = 58): 18.2 (13.8–18.6)	NR	NR
Zhao 2018 [38]	Retrospective observational	NSCLC patients with Exon 20ins *EGFR* mutations (84)	NR	65 (subgroup)	CT	NR	5.7	28.90%
**Second- or later-line treatment**								
Chen 2016 [23]	Prospective observational	Advanced NSCLC with Exon 20 mutation (62)	Zhejiang Cancer Hospital	29 (subgroup)	TKIs (erlotinib, gefitinib, or icotinib)	12.9	1.9	6.9% (PR only)
Wang 2019 [37]	Retrospective observational	Patients with *EGFR* Exon 20ins mutated NSCLC (165)	99 hospitals in 26 different regions in China	165	Platinum-based CT or *EGFR* TKIs (all generations)	NR	Paclitaxel-based CT (n = 34): 4.1 (3.3–4.9) *EGFR* TKIs (n = 18): 2.0 (0.8–3.2; p = 0.342)	NR
DerSarkissian 2019 [10]	Retrospective observational	Relapsed/ refractory patients with advanced/ metastatic NSCLC with *EGFR* Exon 20ins (71)	US	71	CT, *EGFR* TKI, IO	All patients (n = 70): 12.5 (7.5–17.1) *EGFR* TKI only (n = 18): 15.3 (5.0–23.5) Any IO (n = 21): 8.0 (2.0–10.1) CT (n = 17): 17.1 (7.0–30.0)	NR	NR
Chang 2018 [24, 39]	Prospective registry	Heavily pre-treated and refractory advanced NSCLC patients (2242)	10 Asian countries	35 (subgroup)	Afatinib	NR	NR	20% (n = 5)
Janne 2019 [30], Riely 2019 [40], Doebele 2018 [41]	Phase I/II single-arm trial (NCT02716116)	Previously treated advanced NSCLC with *EGFR* Exon 20ins (101)	NR	28 (cohort of interest)	Mobocertinib (TAK-788)	NR	7.3 (4.4–NR)	43% (24–63)
Kim 2019 [42]	Phase II single-arm trial (NCT03414814)	NSCLC patients with *EGFR* Exon 20ins who failed standard CT (15)	Multicenter in South Korea	15	Osimertinib	Not reached (1-year OS rate: 56.3%)	3.5 (1.6–NR)	0%
Piotrowska, 2020a [43]	Phase II single-arm trial (NCT03191149)	Advanced NSCLC with *EGFR* Exon 20ins who received at least one prior line of therapy (21)	Multicenter in US	21	Osimertinib (high dose: 160 mg)	NR	9.6 (4.1–10.7)	24% (n = 17)
Van Veggel 2018 [44]	Retrospective observational	Advanced NSCLC patients with *EGFR* Exon 20ins (17)	4 centers in the Netherlands	17	Osimertinib	NR	3.7 (2.3–5.4)	PR only met: 6%
Heymach 2018 [45]	Phase II single-arm trial (NCT03066206)	Advanced NSCLC with *EGFR* or *HER2* Exon 20 mutation (except *EGFR* T790M) (NR)	US	50 (cohort of interest)	Poziotinib	NR	5.5 (5.2–NA)	Confirmed ORR: 43% (n = 44 evaluated)
Le 2020[46] (ZENITH20-1)	Phase II single-arm trial (NCT03318939)	Advanced NSCLC with *EGFR* Exon 20ins who received at least one prior line of therapy (NR)	Multicenter (US, Canada, Europe)	115 (cohort of interest)	Poziotinib	NR	4.2 (3.7–6.6)	14.8% (8.9–22.6),
Piotrowska 2018 [47]	Phase II single-arm trial (NCT01854034)	Stage IV NSCLC with *EGFR* Exon 20ins (29)	3 hospitals in Boston, MA	29	Luminespib (AUY922)	12.8 (4.5–19.2)	3.3 (1.3–5.6)	19% (PR)
Piotrowska 2015 [48]	Pooled trial analysis (NCT01854034, NCT01922583, and n = 2 from Netherlands compassionate-use program)	Stage IV NSCLC with *EGFR* Exon 20ins (21)	Multicenter (US, Taiwan and the Netherlands)	21	Luminespib (AUY922)	NR	3.9 (2.9–10.7)	24% (PR)
Haura 2019 [49], Cho 2018 [50], Park 2020 [51] (CHRYSALIS)	Phase I single-arm trial (NCT02609776)	Previously treated, advanced NSCLC (116)	Multicenter (South Korea and US)	50	Amivantamab (JNJ-372)	NR	All evaluable (n = 39): 8.3 (3.0–14.8) Post-Platinum-based CT (n = 29): 8.6 (3.7–14.8)	All evaluable (n = 39): 36% (21–53) Post-Platinum-based CT (n = 29): 41% (24–61)
Liu 2020 [52] (RAIN-701)	Phase II single-treatment arm trial (NCT03805841)	Advanced NSCLC (23)	Multicenter (US, Canada, Hong Kong	11 (cohort of interest)	Tarloxotinib	NR	NR	0%
Piotrowska 2020b [53]	Phase I/II single-arm trial (NCT04036682)	NSCLC with documented *EGFR* Exon 20ins (22)	Multicenter (US, Asia, Netherlands)	22	CLN-081	NR	NR	35% (PR)
**Mixed lines of treatment**								
Wu 2019 [32]	Retrospective observational	NSCLC patients with Exon 20ins *EGFR* mutations (84; 59 in 1L cohort)	National Taiwan University Hospital (Taipei, Taiwan)	84 (59 in 1L cohort)	1L CT (n = 43, [24 pemetrexed, 7 taxane-based, 10 gemcitabine-based) TKI-containing (gefitinib, erlotinib or afatinib) (n = 16, [followed by 2L CT: 5 pemetrexed, 3 taxane, 4 gemcitabine; 1 vinorelbine]) 2L TKI (n = 8) 3L+ TKI (n = 15)	1L CT (n = 43): 16.1 (pemetrexed 28 vs. non-pemetrexed 15.4, p = 0.009; taxane 15.9 vs. non-taxane 16.9, p = 0.287; gemcitabine 6.3 vs. non-gemcitabine 18.9, p = 0.003) 1L TKI-containing (n = 16, includes 13 patients who received 2L CT): mOS: 16.8 (vs. 16.1 chemotherapy, p = 0.941)	1L CT (n = 43): 4.2 (pemetrexed 6.2 vs. non-pemetrexed 2.7, p = <0.001; taxane 3.4 vs. non-taxane 3.4, p = 0.361; gemcitabine 3.4 vs. non-gemcitabine 2.2, p = 0.297) 1L TKI-containing (n = 16, includes 13 patients who received 2L CT): 1.8 (vs. 4.2 chemotherapy, p = <0.001) Any TKI (n = 39, [n = 16 1L, n = 8 2L, n = 15 ≥3L]): 4.8	1L pemetrexed CT: 29% (7/24) 1L TKI-containing: 6.3% Any TKI, 1L-≥3L: 5.1% (2/39)
Udagawa 2019 [29]	Retrospective observational	Stage III-IV non-squamous cell NSCLC with *EGFR* or *HER2* Exon 20ins (128; 39 in 1L cohort)	National genome screening project (LC-SCRUM-Japan)	73 (subgroup)	Platinum-based CT, docetaxel containing regiments, PD-1 inhibitor or 1st/2nd generation TKIs	1L platinum-based CT (n = 39): 22.4 (15.3–36.8)	1L platinum-based CT (n = 39): 5.3 ≥2L docetaxel alone, (n = 12): 4.8 ≥2L docetaxel + ramucirumab, (n = 12): 4.7 ≥2nd line PD-1/L1, (n = 21): 3.3 *EGFR* TKI (n = 25): 2.0 (1.4–3.1)	ORR 1L platinum-based CT (n = 39): 23% ≥2L docetaxel alone, (n = 12): 17% ≥2L docetaxel + ramucirumab, (n = 12): 42% ≥2nd line PD-1/L1, (n = 21): 0%
Wu 2011 [26]	Retrospective observational	NSCLC with uncommon *EGFR* Exon 20 mutation (1262)	Taiwan	11 (subgroup)	Gefitinib/ erlotinib	4.80	1.40	0%
Kuiper 2016 [20]	Retrospective observational	NSCLC and Exon 20 and other uncommon *EGFR* mutations (240)	The Netherlands	22 (subgroup)	Erlotinib (93.8%) or Gefitinib (6.3%)	9.7 (0.0–21.1)	2.9 (2.3–3.6)	0%
Tu 2017 [27]	Retrospective observational	NSCLC with uncommon *EGFR* mutation (5363)	China	67	Gefitinib/ erlotinib	12.5 (0.0–25.5)	3.0 (1.3–4.7)	0%
Ovcaricek 2012 [54]	Retrospective observational	Adenocarcinoma or NSCLC NOS (803)	NR	21 (subgroup)	TKIs (erlotinib or gefitinib)	NR	2.5	0/11
Byeon 2019 [55]	Retrospective observational	NSCLC patients with activating *EGFR* mutations 3539 (1479 with advanced NSCLC)	Korea	56 (27 advanced disease)	Platinum-based CT (81.5%); *EGFR* TKI (erlotinib, afatinib, or osimertinib) (22.5%)	*EGFR* TKI: 29.4 (9.3–49.6) All treatments: 29.4 (9.3–49.6)	Platinum-based CT (n = 22): 4.2 (1.7–6.6) *EGFR* TKI: 2.6 (0.7–11.4)	Platinum-based CT (n = 22): 50.0% *EGFR* TKI (n = 4): 25%
Naidoo 2015 [11]	Retrospective observational	Stage IV lung adenocarcinomas with *EGFR* Exon 20ins (1882)	Memorial Sloan-Kettering Cancer Center (New York, NY)	46 (subgroup)	Erlotinib, CT or supportive care	26	NR	Platinum doublet CT: 63% (n = 22/35) Single agent CT: 32% (n = 7/22) Docetaxel = 30%, (n = 3/10) Gemcitabine = 22% (n = 2/9) Immunotherapy: 50% (n = 2/4) Cetuximab: 0% (n = 0/1)
Arcila 2013 [4]	Retrospective observational	Lung adenocarcinomas undergoing routine *EGFR* and *KRAS* testing (600 tested for Exon 20ins)	Memorial Sloan-Kettering Cancer Center (New York, NY)	33	Erlotinib with or without CT (n = 5) CT combo regimens: cisplatin or carboplatin with a taxane or pemetrexed	Patients with advanced disease (n = 15): >4 years	NR	NR
Noronha 2017 [18]	Retrospective observational	Stage IV NSCLC pts with *EGFR* Exon20 mutation (580)	India	20 (subgroup)	Mixed treatments (1L platinum CT-based or TKI, 2L: CT or TKI)	S768 Ic. 2303 G>T (n = 7): 6.0 (0–16.3) H773_V774insH 2319–2320 (CAC) (n = 4): 12.0 (0.2–23.98) V796_D770insASV (2307–2308) GCCAGCGTG (n = 5): 2.0 (1.1–2.9)	NR	NR
Naidoo 2014 [28]	Retrospective observational	Stage IV NSCLC with *EGFR* Exon 20ins or point mutations in *EGFR* S768 or R776 (77)	Memorial Sloan-Kettering Cancer Center (New York, NY)	55 (subgroup)	NR (8 received erlotinib)	1 year OS: 86%	Patients who received erlotinib: 3 months (range <1–8 months)	NR
Lo 2012 [35]	Retrospective observational	NSCLC pts with *EGFR* Exon 20ins (25)	1 US hospital	25	Erlotinib or gefitinib	19 (n = 22)	3.1 on TKI 6.3 on platinum CT	NR
Pan 2014 [19]	Retrospective observational	Patients with lung adenocarcinoma (1086)	China	31	NR (results presented for TKI group)	NR	NR	NR

Abbreviations: 1L = first line; 2L = second line; 3L = third line; CI = confidence interval; CR = complete response; CT = chemotherapy; *EGFR* = epidermal growth factor receptor; ins = insertion; IO = immuno-oncology therapy; m = mutation; mOS = median overall survival; mPFS = median progression-free survival; NA = not applicable; NOS = not otherwise specified; NR = not reported; NSCLC = non-small cell lung cancer; ORR = objective response rate; OS = overall survival; TKI = tyrosine kinase inhibitor

a—LUX-Lung 2 (Phase II), LUX-Lung 3 and LUX-Lung 6 (both Phase III)

*KM curve available

No comparative studies were identified, but rather, nine single-arm phase I/II trials (range 11–115 patients) [40, 42, 43, 45–47, 49, 52, 53] two prospective cohort studies (29 and 35 patients) [23, 29, 39], two pooled analyses of clinical trials (21 and 23 patients) [21, 48], and 22 retrospective observational studies (range 15–165 patients). Half of the studies (n = 16) reported on subgroups or select cohorts of patients with Exon 20ins from studies with broader study populations, and half were conducted in Asia (n = 14), followed by the US (n = 8). Most studies evaluated populations receiving subsequent lines of therapy (n = 14), followed by mixed lines (n = 13), and first-line treatment (n = 7). Two studies did not report the line of therapy received [28, 35]. The included studies were broadly similar in terms of gender, median age, and histology (most were adenocarcinomas), but varied with regards to performance and smoking status.

#### First-line clinical outcomes

Nine studies reported the clinical effectiveness of various first-line treatment regimens in patients with Exon 20ins [10, 21, 29, 32–34, 36–38]. Overall, platinum- and pemetrexed-based chemotherapies were the most efficacious first-line treatments for patients with Exon 20ins [10, 32, 33, 37]. Median OS ranged from 7.1–16.8 months for TKIs (range 16–27 patients) [10, 32, 34] and 6.3–28 months for chemotherapy (range 10–58 patients) [32]. One study, which included 88 patients receiving a mix of TKI (28.0%) and platinum-based chemotherapy (72%), reported a median OS of 16.4 months [33]. Median PFS and ORR were also more favorable for patients with Exon 20ins receiving chemotherapy (median PFS: 3.4–6.9 months for chemotherapy-based regimens [range 10–105 patients] [29, 32, 33, 37, 38] vs. 1.8–6.4 months for TKIs [range 15–25 patients] [21, 32–34, 37]; ORR: 23%–29% [29, 32, 38] for chemotherapy [range 24–45 patients] vs. 0%–8.7% [21, 32, 34] for TKIs [range 15–43 patients]). None of the studies reported safety outcomes for patients with Exon 20ins.

#### Second- or later-line clinical outcomes

Sixteen studies (reported across 21 publications) reported the clinical efficacy/effectiveness and safety of various subsequent-line treatment regimens in patients with Exon 20ins, but based on very limited sample sizes (range 11–165 patients) [10, 23, 24, 29, 30, 37, 39–44, 45–53].

Two studies reported median OS for patients receiving TKIs in subsequent lines of therapy (29 and 18 patients) [10, 23], ranging from 12.9–15.3 months; one of the studies also reported OS for 17 patients receiving chemotherapy (17.1 months) and 21 patients receiving immunotherapy (8.0 months) [10]. One single-arm phase two trial of an investigational compound, luminespib, reported a median OS of 12.8 months, although this trial included only 29 patients with Exon 20ins [47].

Median PFS ranged from 4.1–4.8 months among patients receiving subsequent chemotherapy (range 12–34 patients) [54, 56] and 1.9–3.7 months among those receiving subsequent TKIs (range 15–29 patients) [23, 37, 42, 44], with a recent single-arm trial of high-dose osimertinib (160 mg) conducted in 21 patients reporting a median PFS of 9.6 months [43]. PD-1/L1 inhibitors (21 patients) and luminespib (29 patients) were both associated with a median PFS of 3.3 months, across two separate studies [29, 47]. In two ongoing single-arm trials, amivantamab showed a median PFS of 8.3 months among 39 patients [51], while the mobocertinib trial reported median PFS of 7.3 months in 28 patients [30].

ORRs for amivantamab [51], mobocertinib [30], and CLN-081 [53] were 41%, 43%, and 35%, respectively. For poziotinib, a single-center, single arm trial conducted in 44 patients from MD Anderson, reported an ORR of 43% [45], while a larger multicenter single-arm trial (ZENITH20-1) conducted in 115 patients reported a much lower ORR of 14.8% [46]. ORRs for TKIs and chemotherapies varied significantly across the included studies, ranging from 0%–20% and 17%–42%, respectively.

Safety outcomes were reported for the second-line trials for luminespib [48], osimertinib [42, 46], amivantamab [51], mobocertinib [30, 40], CLN-081 [53], and poziotinib [46]. The most commonly reported treatment-related adverse events (TRAEs) for luminespib (29 patients) were diarrhea (83%) and ocular toxicity (76%), while hypertension (10%) and hypophosphatemia (7%) were the most frequently reported grade 3+ AEs [47]. For mobocertinib (28 patients), diarrhea (85%) and rash (43%) were the most common TRAEs and diarrhea (26%), hypokalemia, nausea, and stomatitis (7% each) were the most common grade 3+ adverse events (AEs) [30]. Similarly, results from ZENITH20-1 (115 patients) show the most common grade 3 TRAEs associated with poziotinib to be diarrhea (25%), rash (28%), and stomatitis (9%) [46]. CLN-081 (22 patients) was associated with rash (60%), stomatitis (13%), and dry skin (13%), with no serious or grade 3 AEs or AEs leading to discontinuations reported [53]. CHRYSALIS (50 patients), showed amivantamab to be associated with grade 3+ TRAEs in 6% of patients with Exon 20ins, with only one grade 3+ case of diarrhea and no grade 3+ rash reported [51]. Dose reductions were reported for 21%, 25%, 0%, and 4% of patients in the luminespib [47], mobocertinib [40], CLN-021 [53], and amivantamab [49] trials respectively. The Korean Cancer Study Group osimertinib trial reported the most frequent AEs to be nausea and vomiting; 20% each.

#### Mixed-line clinical outcomes

Across the studies reporting on mixed lines of therapies, there was a trend towards greater OS benefit with chemotherapy regimens (26–29.4 months) compared with TKI regimens (4.8–19 months). Only one study reported a median PFS of 4.5 months based on 22 patients, compared to a range of 1.4–3.1 months for TKIs (range 11–67 patients).

None of the mixed-line studies reported safety outcomes.

### Humanistic and economic burden

One study assessed the symptoms and health-related quality of life (HRQoL) impact associated with Exon 20ins mutation by conducting interviews with clinical experts (n = 5) and patients with NSCLC (n = 9/10 with Exon 20ins). Symptoms reported by the clinical experts included shortness of breath, chest pain, bone/other pain, and substantial emotional impacts. The study found that causes of poor HRQoL were frequent disease-related symptoms such as fatigue (90% of patients), pain (70%), shortness of breath (70%), and cough (60%), as well as negative impacts on daily activities including household chores and self-care (60%), social activities (50%), work (50%), and family life (40%).

The SLR did not identify any published economic data on cost or resource use associated with Exon 20ins.

## Discussion

The aim of this SLR was to identify and comprehensively summarize the available evidence on the epidemiologic, clinical, humanistic, and economic burden of the *EGFR* Exon 20ins mutation in adult patients with advanced/metastatic NSCLC. Screening approximately 2,500 unique citations yielded 78 articles meeting the prespecified inclusion criteria, the majority of which reported on frequency of mutation (n = 53), clinical outcomes (n = 36), and prognostic impact (n = 13). Only one abstract reporting the humanistic burden of Exon 20ins was identified, and no eligible economic studies were available. To our knowledge, this SLR provides the most comprehensive assessment of the literature reporting on the burden of the Exon 20ins mutation. Methods to quantitatively synthesize the available evidence via meta-analysis were considered, but differences across the included studies with regards to geography, study design, test methodology, and included patient populations were expected to impact the validity and certainty of findings.

The frequency of the Exon 20ins mutation ranged from 0.1%–4.0% among all patients with NSCLC and 1%–12% among those with *EGFR* mutations. Most publications reporting on frequency of mutation were based on Asian or US-based single-center studies, with substantial variation in the genotyping method used. More than eighty unique *EGFR* Exon 20ins mutations have been identified, but only comprehensive testing methods, such as next generation sequencing have the ability to detect all known and unknown variants. Unfortunately, many of the included studies did not report the method used for *EGFR* Exon 20ins detection or used PCR-based testing that focused only on the most common mutations. Therefore, the frequency of *EGFR* Exon 20ins may have been significantly underestimated. Limited findings were also reported on patient-level factors that impact the frequency of Exon 20ins (i.e., age, smoking status, gender). Further research aimed at better understanding the frequency of other molecular characteristics and co-occurring mutations in patients with Exon 20ins is needed. However, current evidence suggests that *EGFR* Exon 20ins tend to be mutually exclusive with other common NSCLC mutation types including other *EGFR* mutations and mutations in *KRAS*, *BRAF*, *HER2*, *NRAS*, *PIK3CA*, *MAP2K1*/*MEK1*, *AKT*, as well as *ALK* rearrangements [4, 57, 58]. Findings from the SLR also indicated that patients with advanced NSCLC and the Exon 20ins mutation have poorer treatment outcomes compared with patients with other *EGFR* mutations and *EGFR* WT across different therapy options and treatment lines. Patients with Exon 20ins treated with afatinib had the lowest PFS, and OS compared to other uncommon *EGFR* mutations in the first-line setting and the lowest ORR compared to other common/uncommon *EGFR* mutations in the second-line setting. Treatment with TKIs was generally associated with worse outcomes in patients with Exon 20ins compared with other mutations across treatment lines; this included lower PFS than those with Exon 18 and T790M mutations in the first-line setting and lower PFS and OS than those with two other types of Exon 20 mutations in the second-line setting. Patients with Exon 20ins who were treated with chemotherapy had significantly shorter OS and comparable PFS to patients with *EGFR* WT in the first-line setting. The clinical differences between T790M and Exon 20ins mutations in NSCLC were less clear than with the classic *EGFR* mutations.

There was a limited number of comparative studies investigating the efficacy and safety of currently available therapies for patients with Exon 20ins. In the first-line setting, there was a trend towards longer OS and PFS and a higher ORR with chemotherapy regimens compared with TKI regimens. A recent large cohort study of patients with advanced NSCLC with Exon20ins (n = 119) suggested that first-line pemetrexed-based chemotherapy regimens are associated with longer OS and PFS than non-pemetrexed-based chemotherapy in this population [59]. While chemotherapy appeared to be associated with longer OS and lower ORR than TKIs in second- and later-line settings in this SLR, there was no clear trend towards greater PFS benefit with either type of therapy. Recently, an indirect treatment comparison that utilized propensity score modeling to match real-world data from the US Flatiron database to patients in the ongoing phase 1–2 mobocertinib trial [30] showed a significant improvement for mobocertinib compared to current standard of care (median PFS: 7.3 vs. 3.5 months, HR: 0.44 [95% CI 0.22, 0.91]) [60]. The real-world evidence from this study also highlighted a lack of standard of care for subsequent-line treatment of patients with Exon 20ins; reporting a mix of immune-oncologic agents (IOs), TKIs, and chemotherapy [60]. Results from our review showed limited data on the effectiveness and/or safety of IOs in patients with Exon 20ins, however, ongoing research will further assess the impact of PD-1/PD-L1 blockade in these patients, given recent findings that patients with *EGFR* Exon 20ins had increased PD-L1 expression and improved outcomes compared to those with *HER2* Exon 20ins [61]. In addition, several emerging compounds, such as DS-2087b [62] and BLU-945 [63], have demonstrated anti-tumour activity in preclinical studies. It should be noted that most studies identified in this SLR included a small number of patients with Exon 20ins, and there was considerable heterogeneity in the clinical and demographic characteristics of the enrolled patients. Further, included studies differed in their methodology, with only a small number of prospective studies identified.

Despite these limitations, this review clearly highlights the gaps in the literature for the humanistic and economic impact of the Exon 20ins mutation. Preliminary evidence suggests that patients with Exon 20ins experience poor HRQoL and a substantial symptom burden, but there remains a significant gap in our understanding of the humanistic and economic burden on this patient population with a demonstrably poor prognosis.

## Conclusions

Findings of this SLR illustrate a wide range in the frequency of Exon 20ins and highlight the need for a better understanding of mutation drivers, including the impact of various test methodologies. Results in terms of prognostic impact and clinical burden, while mainly based on studies with a low sample size, indicated a high unmet need for novel efficacious therapies for patients with advanced NSCLC and *EGFR* Exon 20ins. These patients have poorer treatment outcomes compared with patients with other *EGFR*m and *EGFR* wild-type across different currently available therapy options and treatment lines. While recent data on clinical outcomes for *EGFR* Exon 20ins-targeting therapies are encouraging, evidence regarding their comparative efficacy and safety versus established therapies (i.e. TKIs and chemotherapies) that are known to be associated with poor outcomes are limited. The SLR also uncovered significant gaps in the evidence with regards to data on the economic and humanistic burden of Exon 20 insertion, highlighting a substantial need for additional evidence generation to better understand this among patients with Exon 20ins-positive NSCLC.

## Supporting information

S1 Checklist PRISMA 2009 checklist. (DOC)

S1 Table Search strategies. (DOCX)

S2 Table Summary of included studies. Abbreviations: 1L = first line; 2L = second line; 3L = third line; CT = chemotherapy; ECOG = Eastern Cooperative Oncology Group; EGFR = epidermal growth factor receptor; Ins = insertion; IO = immuno-oncology therapy; IQR = interquartile range; NR = not reported; NSCLC = non-small cell lung cancer; PS = performance status; SCC = squamous cell carcinoma; SCLC = small cell lung cancer; SD = standard deviation; TKI = tyrosine kinase inhibitor.
(DOCX)
