# Supplementary material for: Epidemiological and clinical burden of EGFR Exon 20 insertion in advanced non-small cell lung cancer: A systematic literature review
Source: PLoS One. 2021 Mar 8;16(3):e0247620. doi: 10.1371/journal.pone.0247620 (PMC7939356; doi:10.1371/journal.pone.0247620)
Supplement: S1 Table — (DOCX) [file pone.0247620.s002.docx]

**Table S1. Search Strategies**

| **Number** | **Search String** | **Hits** |
| --- | --- | --- |
| 1. | exp lung cancer/ or exp Lung Neoplasms/ | 566750 |
| 2. | ((lung and (cancer* or neoplasm* or carcinoma* or malignan* or tumor* or tumour*) and non) or (non-small cell lung or nonsmall cell lung or nsclc)).mp. | 351423 |
| 3. | exp Carcinoma, Non-Small-Cell Lung/ or exp non small cell lung cancer/ | 132508 |
| 4. | non small cell.ti,ab. | 150087 |
| 5. | nsclc.ti,ab. | 110995 |
| 6. | ((egfr or epidermal growth factor receptor) and (exon 20 or exon20 or exon-20)).mp. | 1794 |
| 7. | (cmet or c-met or tyrosine-protein kinase Met or hepatocyte growth factor receptor or HGFR).mp. | 15807 |
| 8. | 1 or 2 or 3 or 4 or 5 | 705418 |
| 9. | 6 or 7 | 17551 |
| 10. | 8 and 9 | 3904 |
| 11. | limit 10 to human | 3415 |
| 12. | limit 11 to humans | 3415 |
| 13. | limit 12 to review | 346 |
| 14. | 12 not 13 | 3069 |
| 15. | remove duplicates from 14 | 2524 |
