# Supplementary material for: Epidemiological and clinical burden of EGFR Exon 20 insertion in advanced non-small cell lung cancer: A systematic literature review
Source: PLoS One. 2021 Mar 8;16(3):e0247620. doi: 10.1371/journal.pone.0247620 (PMC7939356; doi:10.1371/journal.pone.0247620)
Supplement: S2 Table — Abbreviations: 1L = first line; 2L = second line; 3L = third line; CT = chemotherapy; ECOG = Eastern Cooperative Oncology Group; EGFR = epidermal growth factor receptor; Ins = insertion; IO = immuno-oncology therapy; IQR = interquartile range; NR = not reported; NSCLC = non-small cell lung cancer; PS = performance status; SCC = squamous cell carcinoma; SCLC = small cell lung cancer; SD = standard deviation; TKI = tyrosine kinase inhibitor. (DOCX) [file pone.0247620.s003.docx]

**Table S2. Summary of Included Studies**

| **StudyID** | **ID of Related Publication(s)** | **Author Year** | **Citation details (Title, Journal, Volume, Issue, Year)** | **Data Source** | **Frequency of mutation** | **Prognostic Impact** | **Clinical Burden** | **Humanistic Burden** |
| --- | --- | --- | --- | --- | --- | --- | --- | --- |
| 2267 | NA | Bell 2018 | Capturing the Patient Experience for the Treatment of EGFR Exon 20 Mutations in Non-Small Cell Lung Cancer | Abstract; IASLC 2018 |  |  |  | Y |
| 86 | NA | Crossland 2018 | The Frequency and Spectrum of EGFR Exon 20 Insertions in NSCLC: A Global Literature Review | Abstract; IASLC 2018 | Y |  |  |  |
| 90 | NA | Ikemura 2018 | Clinical characterization and in silico drug sensitivity prediction model of rare EGFR mutations in non-small cell lung cancer. Journal of Clinical Oncology. 2018. 36:#pages# | Abstract; ASCO 2018 | Y |  |  |  |
| 500 | NA | Steuer 2016 | Role of race in oncogenic driver prevalence and outcomes in lung adenocarcinoma: Results from the Lung Cancer Mutation Consortium. Cancer. 2016. 122:766-72 | Full-text | Y |  |  |  |
| 618 | NA | Kosaka, 2004 | Mutations of the epidermal growth factor receptor gene in lung cancer: Biological and clinical implications. Cancer Research. 2004. 64:8919-8923 | Full-text | Y |  |  |  |
| 661 | NA | Sasaki 2007 | Exon 20 EGFR mutation statuses in Japanese lung cancer patients. Lung Cancer. 2007. 58:324-328 | Abstract; AACR 2007 | Y |  |  |  |
| 906 | NA | Wu 2011 | Effectiveness of tyrosine kinase inhibitors on "uncommon" epidermal growth factor receptor mutations of unknown clinical significance in non-small cell lung cancer. Clinical Cancer Research. 2011. 17:3812-3821 | Full-text | Y | Y | Y |  |
| 934 | NA | Solomon 2012 | Frequency and spectrum of epidermal growth factor receptor (EGFR) mutations in lung cancer in an Australian testing program. Journal of Thoracic Oncology. 2012. 7:S183-S184 | Abstract; Australian Lung Cancer Conference 2012 | Y |  |  |  |
| 948 | NA | Lo 2012 | Clinical behavior of lung cancers harboring EGFR exon 20 insertions. Journal of Thoracic Oncology. 2012. 7:S206-S207 | Abstracts International Association for the Study of Lung Cancer | Y | Y | Y |  |
| 962 | NA | Ovcaricek 2012 | EGFR exon 20 insertion mutations in NSCLC: Frequency and treatment outcomes with EGFR directed TKIs. Journal of Thoracic Oncology. 2012. 7:S64 | Abstract; ASCO 2012 |  |  | Y |  |
| 984 | NA | Arcila 2012 | EGFR exon 20 insertion mutations: Incidence and clinicopathologic characteristics in U.S. patients with lung adenocarcinoma. Journal of Clinical Oncology. 2012. 30. | Abstract; ASCO 2012 | Y |  |  |  |
| 1217 | NA | Skov 2013 | Population based study of the pre-advanced of EGFR mutations in non-small cell lung cancer (NSCLC). Journal of Thoracic Oncology. 2013. 8:S1082-S1083. | Abstract; ASCO 2013 | Y |  |  |  |
| 1244 | NA | Arcila 2013 | EGFR exon 20 insertion mutations in lung adenocarcinomas: Prevalence, molecular heterogeneity, and clinicopathologic characteristics. Molecular Cancer Therapeutics. 2013. 12:220-229 | Full-text | Y |  | Y |  |
| 1248 | NA | Oxnard 2013 | Natural history and molecular characteristics of lung cancers harboring EGFR exon 20 insertions. Journal of Thoracic Oncology. 2013. 8:179-184 | Full-text | Y |  |  |  |
| 1262 | NA | Lai 2013 | EGFR mutations in surgically resected fresh specimens from 697 consecutive Chinese patients with non-small cell lung cancer and their relationships with clinical features. International Journal of Molecular Sciences. 2013. 14:24549-24559 | Full-text | Y |  |  |  |
| 1280 | NA | Veldore 2013 | Epidermal growth factor receptor mutation in non-small-cell lung carcinomas: A retrospective analysis of 1036 lung cancer specimens from a network of tertiary cancer care centers in India. Indian Journal of Cancer. 2013. 50:87-93 | Full-text | Y |  |  |  |
| 1290 | NA | Yasuda 201 | Structural, biochemical, and clinical characterization of epidermal growth factor receptor (EGFR) exon 20 insertion mutations in lung cancer. Sci Transl Med 5(216): 216ra177 | Full-text |  |  | Y |  |
| 1317 | NA | Naidoo 2014 | Exon 20 mutations of the epidermal growth factor receptor (EGFR) in lung adenocarcinomas: Clinicopathologic factors and response to therapy | Abstract; ESMO, IASLC 2014 | Y | Y | Y |  |
| 1338 | NA | Cavic 2014 | High incidence of EGFR gene mutations in Serbian female lung adenocarcinoma patients. European Journal of Cancer. 2014. 50:S143 | Abstract; EACR 2014 | Y |  |  |  |
| 1348 | NA | Farooqi 201 | Characterization of EGFR exon 18 and 20 mutations in NSCLC. Journal of Molecular Diagnostics. 2014. 16:757 | Abstract AMP 2014 | Y |  |  |  |
| 1430 | NA | Beau-Faller 2014 | Rare EGFR exon 18 and exon 20 mutations in non-small-cell lung cancer on 10 117 patients: A multicentre observational study by the French ERMETIC-IFCT network. Annals of Oncology. 2014. 25:126-131 | Full-text | Y |  |  |  |
| 1445 | NA | Weber 2014 | EGFR mutation frequency and effectiveness of erlotinib: A prospective observational study in Danish patients with non-small cell lung cancer. Lung Cancer. 2014. 83:224-230 | Full-text | Y |  |  |  |
| 1495 | NA | Jankovic 2015 | EGFR mutations in advanced non-small cell lung cancer patients in Serbia: 3 year-experience of central testing and gefitinib therapy response. | Abstract; ASCO 2015 | Y |  |  |  |
| 1502 | NA | De La Torre 2015 | Frequency of EGFR mutations in Peruvian patients with non-small cell lung cancer. Journal of Clinical Oncology. 2015. 33:#pages# | Abstract; ASCO 2015 | Y |  |  |  |
| 1565 | NA | Joy Philip 2015 | Is EGFR EXON20 mutation a prognostic/predictive biomarker in our lung cancer patients?. Journal of Thoracic Oncology. 2015. 10:S177 | Abstract; IASLC 2015 | Y |  |  |  |
| 1570 | NA | Domingues 2015 | Clinical outcomes in patients with non-small-cell lung cancer (NSCLC) harboring rare epidermal growth factor receptor (EGFR) mutations. Journal of Thoracic Oncology. 2015. 10:S206-S207 | Abstract; IASLC 2015 | Y |  |  |  |
| 1579 | NA | Pesek 2015 | Rare and common EGFR mutations in patients with advanced NSCLC treated with EGFR-TKIs: A registry-based study. Journal of Thoracic Oncology. 2015. 10:S318 | Abstract; IASLC 2015 | Y |  |  |  |
| 1591 | 2427 | Piotrowska 2015 | Activity of AUY922 in NSCLC patients with EGFR exon 20 insertions. Journal of Thoracic Oncology. 2015. 10:S373-S374 | Abstract; ASCO 2015 |  |  | Y |  |
| 1603 | NA | De La Torre 2015 | Activating and resistance mutations of EGFR in Peruvian patients with metastatic NSCLC. Journal of Thoracic Oncology. 2015. 10:S588 | Abstract; IASLC 2015 | Y |  |  |  |
| 1645 | NA | Chatziandreou 2015 | Comprehensive molecular analysis of NSCLC; Clinicopathological associations. PLoS ONE. 2015. 10:e0133859 | Full-text | Y |  |  |  |
| 1650 | NA | Jain 2015 | Impact of smoking and brain metastasis on outcomes of advanced EGFR mutation lung adenocarcinoma patients treated with first line epidermal growth factor receptor tyrosine kinase inhibitors. PLoS ONE. 2015. 10:e0123587 | Full-text | Y |  |  |  |
| 1671 | NA | Yang 2015 | Clinical activity of afatinib in patients with advanced non-small-cell lung cancer harbouring uncommon EGFR mutations: A combined post-hoc analysis of LUX-Lung 2, LUX-Lung 3, and LUX-Lung 6. The Lancet Oncology. 2015. 16:830-838 | Full-text |  | Y | Y |  |
| 1696 | NA | Naidoo 2015 | Epidermal growth factor receptor exon 20 insertions in advanced lung adenocarcinomas: Clinical outcomes and response to erlotinib. Cancer. 2015. 121:3212-3220 | Full-text | Y | Y | Y |  |
| 1706 | NA | Heigener 2015 | Afatinib in Non-Small Cell Lung Cancer Harboring Uncommon EGFR Mutations Pretreated With Reversible EGFR Inhibitors. Oncologist. 2015. 20:1167-1174 | Full-text | Y |  |  |  |
| 1716 | NA | Han 2016 | Association of EGFR mutation subtypes with clinical and demographic characteristics of patients (pts) with aNSCLC: IGNITE and ASSESS pooled analysis. Journal of Thoracic Oncology. 2016. 11:S79-S80 | Abstract; ESMO 2016 | Y |  | Y |  |
| 1806 | NA | Chen 2016 | Clinical efficacy of first-generation EGFR-TKIs in patients with advanced non-small-cell lung cancer harboring EGFR exon 20 mutations. OncoTargets and Therapy. 2016. 9:4181-4186 | Full-text | Y | Y | Y |  |
| 1847 | NA | Kuiper 2016 | Non-classic EGFR mutations in a cohort of Dutch EGFR-mutated NSCLC patients and outcomes following EGFR-TKI treatment. British Journal of Cancer. 2016. 115:1504-1512 | Full-text | Y | Y | Y |  |
| 1916 | NA | Lam 2017 | Circulating tumor DNA (ctDNA)-based genomic profiling of known cancer genes in lung squamous cell carcinoma (LUSC). Journal of Thoracic Oncology. 2017. 12:S947-S948 | Abstract; IASLC 2018 | Y |  |  |  |
| 1944 | 2078 | Tu 2017 | The predictive value of uncommon EGFR mutation in patients with non-small-cell lung cancer. Journal of Clinical Oncology. 2017. 35:#pages# | Abstract; ASCO 2017 | Y |  |  |  |
| 1954 | NA | Mas 2017 | Prevalence of EGFR mutations in the Peruvian population: Study in a large cohort of patients with NSCLC. Journal of Clinical Oncology. 2017. 35:#pages# | Abstract; ASCO 2017 | Y |  |  |  |
| 2016 | NA | Noronha 2017 | Epidermal growth factor receptor exon 20 mutation in lung cancer: Types, incidence, clinical features and impact on treatment. OncoTargets and Therapy. 2017. 10:2903-2908 | Full-text | Y |  |  |  |
| 2060 | NA | Shen 2017 | Comparing the effects of afatinib with gefitinib or Erlotinib in patients with advanced-stage lung adenocarcinoma harboring non-classical epidermal growth factor receptor mutations. Lung Cancer. 2017. 110:56-62 | Full-text | Y |  |  |  |
| 2078 | 1944 | Tu 2017 | A comprehensive review of uncommon EGFR mutations in patients with non-small cell lung cancer. Lung Cancer. 2017. 114:96-102 | Full-text | Y | Y | Y |  |
| 2079 | NA | Leduc 2017 | Clinical and molecular characteristics of non-small-cell lung cancer (NSCLC) harboring EGFR mutation: Results of the nationwide French Cooperative Thoracic Intergroup (IFCT) program. Annals of Oncology. 2017. 28:2715-2724 | Full-text | Y |  |  |  |
| 2101 | NA | Cheng 2017 | A multicenter, non-interventional study on real world EGFR testing and in patients with IIIB/IV NSCLC in Northern China. Journal of Thoracic Oncology. 2017. 12:S1734-S1735 | Abstract; IASLC 2017 | Y |  |  |  |
| 2115 | NA | Cardona 2017 | EGFR exon 20 insertions in lung adenocarcinomas: Molecular and clinicopathologic characteristics among Hispanics (geno1.2-clicap). Journal of Thoracic Oncology. 2017. 12:S2260-S2261 | Abstract; IASLC 2017 | Y |  |  |  |
| 2249 | NA | Ferreira 2018 | Updated EGFR Mutation Frequency in 1,689 NSCLC Brazilian Patients - A National-Wide Study. Journal of Thoracic Oncology. 2018. 13:S676 | Abstract; IASLC 2018 | Y |  |  |  |
| 2260 | NA | Zhao 2018 | EGFR exon20 Insertion Patients Treated with First-Line Chemotherapy in Non-Small Cell Lung Cancer. Journal of Thoracic Oncology. 2018. 13:S507 | Abstract; ASCO 2018 |  |  | Y |  |
| 2274 | 2296 | Chang 2018 | Named Patient Use Program for Afatinib in Advanced NSCLC with Progression on Prior Therapy: Experience from Asian Centers. Journal of Thoracic Oncology. 2018. 13:S463 | Abstract and Slide Deck; WCLC 2018 |  | Y | Y |  |
| 2279 | NA | Wang 2018 | Real World EGFR Mutation Profile from 1699 Non-Small Cell Lung Cancer Patients in Eastern China | Abstract; IASLC 2018 | Y |  |  |  |
| 2289 | NA | Cardona 2018 | EGFR exon 20 insertion in lung adenocarcinomas among Hispanics (geno1.2-CLICaP). Lung Cancer. 2018. 125:265-272 | Full-text | Y | Y | Y |  |
| 2296 | 2274 | Chang 2018 | Afatinib Named Patient Use Program in Advanced NSCLC with Progression on Prior Therapy: Experience from Asian Centers. Journal of Thoracic Oncology. 2018. 13:S1056-S1057 | Abstract; WCLC 2018 |  |  | Y |  |
| 2303 | NA | Riess 2018 | Diverse EGFR Exon 20 Insertions and Co-Occurring Molecular Alterations Identified by Comprehensive Genomic Profiling of NSCLC | Abstract; IASLC 2018 | Y |  |  |  |
| 2310 | NA | Pan 2014 | Prevalence, Clinicopathologic Characteristics, and Molecular Associations of EGFR Exon 20 Insertion Mutations in East Asian Patients with Lung Adenocarcinoma. Annals of Surgical Oncology. 2014. #volume#:1-7 | Full-text | Y | Y | Y |  |
| 2336 | NA | Wu 2019 | Effectiveness of treatments in advanced nonsmall cell lung cancer with Exon 20 insertion epidermal growth factor receptor mutations. Respirology. 2018. 23:168 | Full-text |  | Y | Y |  |
| 2351 | 2501 | Byeon 2018 | Clinical outcomes of EGFR exon 20 insertion in advanced NSCLC in Korea. Journal of Clinical Oncology. 2018. 36:#pages# | Abstract; ASCO 2018 | Y |  |  |  |
| 2358 | ASCO-1, WL1 | Doebele 2018 | First report of safety, PK, and preliminary antitumor activity of the oral EGFR/HER2 exon 20 inhibitor TAK-788 (AP32788) in non-small cell lung cancer (NSCLC). Journal of Clinical Oncology. 2018. 36:#pages# | Abstract; ASCO 2018 |  |  | Y |  |
| 2426 | NA | Shapochka 2018 | EGFR and ALK Mutation Status in Advanced Lung Adenocarcinomas: Prevalence, Gender and Age-related Peculiarities Among Patients in Ukraine | Abstract; ESMO 2018 | Y |  |  |  |
| 2427 | 1591 | Piotrowska 2018 | Activity of the Hsp90 inhibitor luminespib among non-small-cell lung cancers harboring EGFR exon 20 insertions. Annals of Oncology. 2018. 29:2092-2097 | Full-text |  |  | Y |  |
| 2435 | NA | Kate 2019 | Outcome of uncommon EGFR mutation positive newly diagnosed advanced non-small cell lung cancer patients: a single center retrospective analysis. Lung Cancer: Targets and Therapy. 2019. 10:1-10 | Full-text | Y | Y | Y |  |
| 2444 | NA | Fang 2019 | EGFR exon 20 insertion mutations and response to osimertinib in non-small-cell lung cancer. BMC Cancer. 2019. 19:595 | Full-text | Y |  | Y |  |
| 2459 | 2477 | Chantharasamee 2018 | The characteristics and clinical outcome of metastatic NSCLC harboring uncommon EGFR mutation at Thailand’s tertiary referral center. Annals of Oncology. 2018. 29. | Abstract; ESMO 2018 | Y |  |  |  |
| 2461 | NA | Van Veggel 2018 | Osimertinib treatment for patients with EGFR exon 20 insertion positive non-small cell lung cancer. Annals of Oncology. 2018. 29:#pages# | Abstract; ESMO 2018 |  |  | Y |  |
| 2462 | ASCO-3 | Cho 2018 | JNJ-61186372 (JNJ-372), an EGFR-cMET bispecific antibody, in advanced non-small cell lung cancer (NSCLC): An update on phase I results. Annals of Oncology. 2018. 29:#pages# | Abstract and Poster; ESMO 2018 |  |  | Y |  |
| 2477 | 2459 | Chantharasamee 2019 | Clinical outcome of treatment of metastatic non-small cell lung cancer in patients harboring uncommon EGFR mutation. BMC Cancer. 2019. 19:701 | Full-text | Y |  |  |  |
| 2501 | 2351 | Byeon 2019 | Clinical Outcomes of EGFR Exon 20 Insertion Mutations in Advanced Non-small Cell Lung Cancer in Korea. Cancer Research and Treatment. 2019. 51:623-631 | Full-text | Y |  | Y |  |
| ASCO-1 | 2358, WL1 | Janne 2019 | Antitumor activity of TAK-788 in NSCLC with EGFR exon 20 insertions. Journal of Clinical Oncology 37, no. 15_suppl (May 20, 2019) 9007-9007. | Abstract; ASCO 2019 |  |  | Y |  |
| ASCO-3 | 2284 | Haura 2019 | JNJ-61186372 (JNJ-372), an EGFR-cMet bispecific antibody, in EGFR-driven advanced non-small cell lung cancer (NSCLC). Abs 9009 | Abstract and Poster; ASCO 2019 |  |  | Y |  |
| ESMO-1 | NA | Kim 2019 | Phase II study of osimertinib in NSCLC patients with EGFR exon 20 insertion mutation: A multicenter trial of the Korean Cancer Study Group (LU17-19), Annals of Oncology, Volume 30, Issue Supplement_5, October 2019, mdz260.051 | Abstract; ESMO 2019 |  |  | Y |  |
| WL1 | ASCO-1, 2358 | Riely 2019 | P1.01-127 - Antitumor Activity of the Oral EGFR/HER2 Inhibitor TAK-788 in NSCLC with EGFR Exon 20 Insertions | Abstract and Poster; WCLC 2019 |  |  | Y |  |
| WL2 | NA | Udagawa 2019 | OA07.03 - Clinical Outcome of Non-Small Cell Lung Cancer with EGFR/HER2 Exon 20 Insertions Identified in the LC-SCRUM-Japan | Abstract; WCLC 2019 |  |  | Y |  |
| WL3 | NA | DerSarkissian 2019 | P2.01-103 - Real-World Treatment Patterns and Survival in Non-Small Cell Lung Cancer Patients with EGFR Exon 20 Insertion Mutations | Abstract; WCLC 2019 |  |  | Y |  |
| WL4 | NA | Heymach 2018 | Heymach et al. A Phase II Trial of Poziotinib in EGFR and HER2 exon 20 Mutant Non-Small Cell Lung Cancer (NSCLC). OA02.06 | Abstract; WCLC 2018 |  |  | Y |  |
| ASCO-4 | NA | Le 2020 | Poziotinib shows activity and durability of responses in subgroups of previously treated EGFR exon 20 patients. [Abs 9514](https://meetinglibrary.asco.org/record/184819/abstract) | Abstract and Poster, ASCO 2020 |  |  | Y |  |
| ASCO-5 | NA | Piotrowska 2020 | ECOG-ACRIN 5162: A phase II study of osimertinib 160 mg in NSCLC with EGFR exon 20 insertions. Abs 9513 | Abstract and Poster, ASCO 2020 |  |  | Y |  |
| ASCO-6 | ASCO-3, 2284 | Park 2020 | Amivantamab (JNJ-61186372), an anti-EGFR-MET bispecific antibody, in patients with EGFR exon 20 insertion (exon20ins)-mutated non-small cell lung cancer (NSCLC). Abs 9512 | Abstract and Poster, ASCO 2020 |  |  | Y |  |
| ESMO-2 | NA | Liu 2020 | First analysis of RAIN-701: Study of tarloxotinib in patients with non-small cell lung cancer (NSCLC) EGFR Exon 20 insertion, HER2-activating mutations & other solid tumours with NRG1/ERBB gene fusions. Abs LBA61 | Abstract and Mini-Oral, ESMO 2020 |  |  | Y |  |
| ESMO-3 | NA | Piotrowska 2020 | Preliminary Safety and Activity of CLN-081 in NSCLC with EGFR Exon 20 Insertion Mutations (Ins20) | Abstract and Poster, ESMO 2020 |  |  | Y |  |
